# Revealing the novel effect of Jinghua Weikang capsule against the antibiotic resistance of *Helicobacter pylori*

**DOI:** 10.3389/fmicb.2022.962354

**Published:** 2022-09-06

**Authors:** Xiaofen Jia, Qiuyue Huang, Miaomiao Lin, Yingming Chu, Zongming Shi, Xuezhi Zhang, Hui Ye

**Affiliations:** Department of Integrated Traditional Chinese and Western Medicine, Peking University First Hospital, Institute of Integrated Traditional Chinese and Western Medicine, Peking University, Beijing, China

**Keywords:** *Helicobacter pylori*, antibiotic resistance, metronidazole, biofilm, efflux pump, adhesion, Jinghua Weikang capsule

## Abstract

**Background:**

*Helicobacter pylori* (*H. pylori*) infects half of the human population globally. Eradication rates with triple or quadruple therapy have decreased owing to the increasing rate of antibiotic resistance. Jinghua Weikang capsule (JWC) is the first and most popular Chinese patent medicine approved by the state for the treatment of gastritis and peptic ulcers caused by *H. pylori* infection in China. Previous studies have found that JWC has a certain bactericidal effect on drug-resistant *H. pylori* and its major component, *Chenopodium ambrosioides* L. inhibits biofilm formation, but the mechanism remains unclear. This study focused on drug-resistant *H. pylori* and explored whether JWC could reverse drug resistance and its related mechanisms.

**Method:**

The agar plate dilution method, E-test method, and killing kinetics assay were used to evaluate the bactericidal effect of JWC on antibiotic-resistant *H. pylori* and its effect on antibiotic resistance. Sanger sequencing was used to detect mutations in drug resistance genes. The crystal violet method, scanning electron microscopy, and confocal laser scanning microscopy were used to evaluate the effects of JWC on biofilms. qPCR was performed to evaluate the effect of JWC on the expression of efflux pump-related genes. qPCR and immunofluorescence were used to evaluate the effects of JWC on *H. pylori* adhesion.

**Results:**

JWC showed considerable antibacterial activity against drug-resistant *H. pylori* strains, with minimum inhibitory concentration (MIC) values ranging from 64 to 1,024 μg/ml. The MIC of metronidazole (MTZ) against *H. pylori* 26,695–16R decreased from 64 to 6 μg/ml after treatment with 1/2 MIC of JWC. The resistance of *H. pylori* 26,695–16R to MTZ was reversed by JWC, and its effect was better than that of PaβN and CCCP. *H. pylori* 26,695–16R is a moderate biofilm-forming strain, and JWC (16–64 μg/ml) can inhibit the formation of biofilms in *H. pylori* 26,695–16R. JWC reduced the expression of HP0605-HP0607 (*hefABC*), HP0971-HP0969 (*hefDEF*), HP1327-HP1329 (*hefGHI*), and HP1489-HP1487. JWC reduced the adhesion of *H. pylori* to GES-1 cells and the expression of adhesives *NapA*, *SabA*, and *BabA*.

**Conclusion:**

The reversal of MTZ resistance by JWC may be achieved through the adhesin/efflux pump-biofilm pathway.

## Introduction

*Helicobacter pylori* (*H. pylori*) infects half of the global population and can cause a variety of gastric diseases, such as peptic ulcers, chronic gastritis, gastric cancer, and extragastric diseases (Zamani et al., 2018; Ren et al., 2022). The eradication of *H. pylori* has significantly reduced the incidence and mortality rates of gastric cancer (Chiang et al., 2021). However, large-scale eradication has led to increasing rates of *H. pylori* resistance to multiple antibiotics, the main cause of eradication failure (Zhong et al., 2022). The eradication rate of triple therapy is currently less than 70% (Liu et al., 2018). Since 2017, *H. pylori* has been listed by the World Health Organization as one of the 20 pathogens that pose the most serious threat to human health owing to its drug resistance (Tacconelli et al., 2018). It is difficult to reduce the resistance rate. The causes and mechanisms of antibiotic resistance are complicated and include specific resistance factors against a particular antibiotic (resistance gene mutation; Tshibangu-Kabamba and Yamaoka, 2021), as well as nonspecific resistance factors, such as biofilms and efflux pumps (Zanotti and Cendron, 2019). Bacterial biofilms are complexes composed of bacteria and extracellular polymers (EPS) such as proteins, polysaccharides, lipids, and DNA secreted by bacteria, which create a protective environment for bacteria (Høiby et al., 2010; Rather et al., 2021). Bacteria that form biofilm structures are highly resistant to harsh external environments such as antibiotic exposure. It has been demonstrated that bacteria are 10–1,000 times more resistant to antibiotics when they form biofilms (Chen and Wen, 2011; Yonezawa et al., 2019; Hou et al., 2022). The formation of *H. pylori* biofilms includes four steps: adhesion, growth, maturation, and diffusion (Hou et al., 2022). Adhesion is the first step and a prerequisite for biofilm formation. The adhesion of *H. pylori* is mediated by dozens of specific adhesin receptors, among which blood group antigen-binding adhesin (*BabA*), sialic acid adhesin (*SabA*), and neutrophil-activating protein A (*NapA*) play major roles (Fu, 2014; Doohan et al., 2021; Matos et al., 2021). The bacterial efflux pump is a transmembrane transporter protein that mediates the pumping of intracellular drugs out of the cell, thereby reducing the intracellular drug concentration and promoting drug resistance. The efflux effect of the active efflux pump system in bacteria is an important mechanism underlying nonspecific drug resistance. The resistance nodulation and cell division (RND) family, major facilitator super (MFS) family, and ATP-binding cassette (ABC) family are the predominant efflux pump families in *H. pylori.* Several studies have shown that the efflux pump expression in biofilm-forming bacteria is higher than that in planktonic cells (Soto, 2013). The expression levels of HP0605–HP0607 (*hefABC*), HP0971-HP0969 (*hefDEF*), HP1327–HP1329 (*hefGHI*), and HP1489–HP1487 in biofilm-forming strains are higher than those in planktonic bacteria (Yonezawa et al., 2019). The expression level of Hp1174 [glucose/galactose transporter (*gluP*)] also follows these rules (Ge et al., 2018). This suggests that efflux pumps and biofilms may interact or act synergistically to increase drug resistance.

As an exogenous pathogenic factor, *H. pylori* is equivalent to “evil *qi*” in traditional Chinese medicine (TCM). According to TCM, *H. pylori* infection mostly presents with basic symptoms of cold and heat in complexity and deficiency in complexity. Jinghua Weikang capsules (JWC) were obtained from *Chenopodium ambrosioides L.* (CAL) and Rubiaceae adina pilulifera (RAP). CAL regulates *qi*, disperses cold, and kills insects, while RAP clears heat and removes blood stasis. The two are used in combination to harmonize the spleen and stomach. It is widely used in digestive diseases related to *H. pylori* infection and has a good basis for application and clinical efficacy (Hui and Xuezhi, 2014). Previous studies have found that JWC and its main component, CAL, can kill and inhibit standard drug-resistant *H. pylori*, and CAL can inhibit the formation of drug-resistant *H. pylori* biofilms (Liu et al., 2013; Ye et al., 2015; Enen et al., 2020). In the remedial treatment of patients with chronic gastritis or peptic ulcer suffering from *H. pylori* infection relapses, the addition of JWC improved the eradication rate of *H. pylori* compared to bismuth quadruple therapy (90.0 *vs*. 82.0%; Hong et al., 2016). Antibiotic resistance is the main reason for *H. pylori* eradication failure. JWC can contribute toward improving the eradication rate of *H. pylori* in remedial treatment, however, its mechanism is still unknown. This study explores whether JWC can reverse the drug resistance and related mechanisms in *H. pylori*.

## Materials and methods

### Drug preparation

The volatile oil of JWC (Tasly Pharmaceutical Group Co. LTD, Tianjin, China) was mixed with dimethyl sulfoxide (DMSO, Thermo Fisher Scientific, Waltham, MA, United States) at a 1:4 volume ratio and dissolved in sterile deionized water. The density of JWC was 937 mg/ml.

### Bacterial culture and identification

The *H. pylori* strains used in this experiment (Table 1) were all obtained from the Department of Gastroenterology, Peking University First Hospital, among which *H. pylori* Strains1–6 were isolated from clinical patients who had previously failed *H. pylori* eradication therapy and required drug sensitivity test to guide the eradication regimen and *H. pylori* 26,695–16R is an *rdxA* null deletion mutant derivative of *H. pylori* 26,695 (Sisson et al., 2000). *H. pylori* strains were frozen in –80°C refrigerator. The cryopreservation solution was prepared by brain heart infusion (OXOID, Basingstoke, United Kingdom) and glycerol (Solarbio, Beijing, China). *H. pylori* was inoculated on Columbia blood agar (OXOID, Basingstoke, United Kingdom) plates containing 8% sheep blood (Lablead, Beijing, China), placed upside down at 37°C in a microaerophilic (85% N_2_, 10% CO_2_, 5% O_2_) environment for 48–72 h, and the positive ones were sub-cultured, generally no more than seven generations. The strains were identified by colony morphology, Gram staining, and rapid urease tests.

**Table 1:** The primers used in Sanger sequencing.

Primer name	Sequence (5′→3′)	References
*rdxA* fwd	ATGGTAATTGTTTCGTTAGGG	Teh et al. (2014)
*rdxA* rev	CTCCTTGAACTTTAATTTAG	Teh et al. (2014)
*frxA* fwd	TGGATATGGCAGCCGTTTA	Teh et al. (2014)
*frxA* rev	GGTTATCAAAAAGCTAACAGCG	Teh et al. (2014)
23S rRNA fwd	CCACAGCGATGTGGTCTCAG	Teh et al. (2014)
23S rRNA rev	CTCCATAAGA GCCAAAGCCC	Teh et al. (2014)
*gyrA* fwd	AGCTTATTCCATGAGCGTGA	Teh et al. (2014)
*gyrA* rev	TCAGGCCCTTTGACAA ATTC	Teh et al. (2014)
*gyrB* fwd	CCCTAACGAAGCCAAAATCA	Teh et al. (2014)
*gyrB* rev	GGGCGCAAATAACG ATAGAA	Teh et al. (2014)

### Drug susceptibility test

#### E-test method

*Helicobacter pylori* cultured for 48–72 h was uniformly ground into a cryopreservation solution, and the bacterial solution was diluted to 3 × 10^8^ CFU/ml. One hundred microliters of bacterial solution was pipetted onto the surface of the medium, smeared evenly with L sticks, placed on an E-test drug susceptibility test strip (Liofilchem, Roseto degli Abruzzi, Italy), and incubated at 37°C for 72 h in a microaerophilic environment to read the results. The value corresponding to the ring region where the bacteria stops growing is the minimum inhibitory concentration (MIC) of an antibiotic for *H. pylori.* According to EUCAST Clinical Breakpoint standard 2022, *H. pylori* strains that could grow in medium containing amoxicillin (MIC > 0.125 μg/ml), levofloxacin (MIC > 1 μg/ml), clarithromycin (MIC > 0.5 μg/ml), and metronidazole (MIC > 8 μg/ml) were identified as drug-resistant strains. Phenylalanine-arginine β-naphthylamide (PaβN, Sigma-Aldrich, St. Louis, MO, United States) and carbonyl cyanide m-chlorophenylhydrazonequinoline (CCCP, Sigma-Aldrich, St. Louis, MO, United States) are the most common inhibitors of efflux pumps and have also been found to inhibit biofilm formation (Zhang et al., 2010; Kinana et al., 2016; Tang et al., 2020; Dawan et al., 2022).

#### Agar plate dilution method

Media containing different concentrations of JWC (2048, 1,024, 512, 256, 128, 64, 32, and 16 μg/ml) were prepared. Drug-free and DMSO (Sigma-Aldrich, St. Louis, MO, United States) -containing media were used as controls. *H. pylori* cultured for 48–72 h was uniformly ground into the cryopreservation solution, and the bacterial solution was diluted to 3 × 10^8^ cfu/ml. A 1 μl sterile inoculating ring was used to inoculate the bacterial solution on the surface of the drug-containing medium, and observed after 72 h of culture in a microaerobic environment at 37°C. The MIC of the lowest drug concentration on a medium without colonies was the MIC for JWC.

### Inhibiting kinetics and killing kinetics assay

The inhibition and killing kinetics assays were performed as previously reported (Shen et al., 2021; Peng et al., 2022). For the inhibition kinetics assay, *H. pylori* 26,695–16R cultured for 48–72 h was collected at 0 (control), 0.25, 0.5, and 1 times the MIC concentration for JWC in Brucella Broth (BD, Franklin Lakes, NJ, United States) containing 10% Foetal Bovine Serum (FBS, BI, Kibbutz Beit Haemek, Israel) and shaken (100–120 rpm) at 37°C. Then, at 0, 12, 24, 36, 46, 48, 60, and 72 h, 100 μl of each sample was tested for absorbance at 600 nm. Three holes were set in each sample, and the experiment was repeated three times. For the killing kinetics assay, *H. pylori* 26,695–16R cultured for 48–72 h was collected at 0 (control), 1, 2, and 4 times the MIC concentration of JWC in Brucella broth containing 10% FBS and treated in a shaker (100–120 rpm) at 37°C. At 0, 4, 8, 12, and 24 h, 30 μl was removed from each sample, and serial 10-fold dilutions were prepared in Brucella broth. One hundred microliters of the diluted solution were plated on Columbia blood agar plates and incubated at 37°C, and colonies were counted and averaged after 3 days. The results are expressed as Log_10_ (CFU/ml). This experiment was repeated twice.

### Sanger sequencing

Bacterial genomic DNA was extracted using the QIAamp DNA Mini Kit (QIAGEN GmbH, Hilden, Germany), and the samples were stored at –80°C. The *23S rRNA*, *gyrA* (hp0701), *gyrB* (hp0501), *rdxA* (hp0954), and *frxA* (hp0642) fragments were amplified using PCR. Primers used are listed in Table 1 (Teh et al., 2014). PCR amplification products were examined on 1.0% agarose gels and bands were observed. The PCR product was separated and purified using magnetic beads (Ensure Biologicals, Shanghai, China). The PCR product sequencing was performed by Beijing Liuhe Bgi Co. Ltd. (Beijing, China).

### Quantitative real-time PCR

*Helicobacter pylori* cultured for 48–72 h was collected in Brucella broth containing different concentrations of drugs and shaken (100–120 rpm) at 37°C for 2 h. Total RNA of *H. pylori* was extracted using the TRIzol method, mRNA was reverse transcribed into cDNA using a High Capacity cDNA Reverse Transcription Kit (Thermo Fisher Scientific, Waltham, MA, United States), and PCR amplification was carried out using PowerUp^™^ SYBR^™^Green Master Mix (Thermo Fisher Scientific, Waltham, MA, United States). Primers used in this experiment are listed in Table 2 (Yonezawa et al., 2019; Zhong et al., 2021).

**Table 2:** Primers sequences for qPCR.

Primer name	Sequence (5′→3′)	References
16S rRNA fwd	GGGTGAGTAACGCATAGGTCA	Designed for this study
16S rRNA rev	TTTACGCCCAGTGATTCCGA	Designed for this study
HP0605 fwd	AGCGCAAGAACTCAGTGTCA	Zhong et al. (2021)
HP0605 rev	GCTTGGAGTTGTTGGGTGTT	Zhong et al. (2021)
HP0971 fwd	TTACCGGCAAAGGGATACG	Yonezawa et al. (2019)
HP0971 rev	AAATTGGATCGCTCGTTGTATG	Yonezawa et al. (2019)
HP1327 fwd	GCCAGGCTTGATGAAGAAAA	Yonezawa et al. (2019)
HP1327 rev	TTAGCCTGCTTGCCGTAAAT	Yonezawa et al. (2019)
HP1489 fwd	TAGGCGCTCAAGTGGCTTAT	Yonezawa et al. (2019)
HP1489 rev	TCAGATCGGGCAGATTTTTC	Yonezawa et al. (2019)
*BabA* fwd	CCCGCGCTCAAAGAAAACAA	Designed for this study
*BabA* rev	GTGGTGGTTACGGTTTTGCC	Designed for this study
*SabA* fwd	TCGTCATCAGTGGCGTTTCA	Designed for this study
*SabA* rev	TCCCTGTAGCTTGAGCTTGC	Designed for this study
*NapA* fwd	TTGGAATGTGAAAGGCACCGATTTT	Designed for this study
*NapA* rev	GCCTTCTTTTTCAGCGGTGTTAGA	Designed for this study

### Crystal violet staining

The biofilm of *H. pylori* was cultured using the 96-well plate method. *H. pylori* cultured for 48–72 h was collected in Brucella broth containing different concentrations of drugs and treated in a microaerobic environment at 37°C for 72 h. Each group was provided with nine holes. After incubation, the upper bacterial solution was gently discarded, the plate was rinsed three times with Phosphate Buffer Saline (PBS, Thermo Fisher Scientific, Waltham, MA, United States), 200 μl of anhydrous methanol (Beijing Tongguang Fine Chemical, Beijing, China) was added to each well for 15 min, the methanol was discarded, and the plate was air-dried. Then, 200 μl of 1% ammonium oxalate crystal violet reagent (Solarbio, Beijing, China) was added to each well for staining for 5 min and then washed with running water. After natural drying, 200 μl 95% ethanol (Beijing Tongguang Fine Chemical, Beijing, China) was added to each well and dissolved in a shaker (80 rpm) at 37°C for 30 min. The Optical Density (OD) value was measured using a microplate reader (TECAN, Männedorf, Switzerland; absorbance at 590 nm). Dc is the OD value of the blank wells and D is the mean value of the remaining OD values after removing the outliers. A value of D > 4 × Dc, was determined to be a strong biofilm-forming *H. pylori* strain; 2 × Dc < D ≤ 4 × Dc, a moderate biofilm-forming *H. pylori* strain; Dc < D ≤ 2 × Dc, a weak biofilm-forming *H. pylori* strain; and D ≤ Dc, a non-biofilm-forming *H. pylori* strain.

### Scanning electron microscope

The nitrocellulose (NC) membrane (GE, Boston, Mass, United States) was cut into 1 × 1 cm pieces to prepare a solid medium containing NC membranes and different concentrations of drugs. *H. pylori* cultured for 48–72 h was uniformly ground into the cryopreservation solution, and the bacterial solution was diluted to 3 × 10^8^ cfu/ml. A total of 10 μl of bacterial solution was pipetted onto the NC membrane, spread evenly, and incubated in a microaerophilic environment at 37°C for 72 h. The NC membrane was then removed and placed in a 6-well plate, and an appropriate amount of glutaraldehyde (Regen Biotechnology Co., LTD, Beijing, China) was added to fix it at 4°C for 2 h. After fixation, the membrane was air-dried and the results were observed using a field emission scanning electron microscope (JEOL, Tokyo, Japan). Metronidazole used in this study was obtained from Aladdin, Shanghai, China.

### Confocal laser scanning microscope

A LIVE/DEAD^™^ *Bac*Light^™^ Bacterial Cell Activity Assay Kit (Thermo Fisher Scientific, Waltham, MA, United States) was used for fluorescence staining. Dye A (SYTO^™^ 9 dye) penetrates the bacterial cell membrane and binds to DNA to stain bacteria green. Dye B (propidium iodide) only penetrates incomplete bacterial cell membranes; when the bacteria die, the permeability of the cell membrane changes, and dye B dyes the dead bacteria red. Glycerin was used for microscopic observations.

The biofilm was constructed as described above, and the incubated NC membrane was removed aseptically, placed in a 24-well plate and rinsed 3 times in PBS. Mix 3 μl A and B 1:1, add 1 ml normal saline, add 100 μl to each well, and incubate in the dark for 15 min. The NC membrane was removed and placed on a glass slide, and glycerol was added for observation under a microscope. The samples were observed within 1 h to avoid the effects of bacterial death caused by prolonged exposure. CLSM (Leica, Wetzlar, Germany) was performed using an argon laser at 488 nm excitation, with the blue channel receiving the green signal and the 560 nm green channel receiving the red signal, and scanning from the free side of the *H. pylori* biofilm to the attached side of the slide layer-by-layer at an interval of 1 μm.

### Immunofluorescent staining

GES-1 cells (1 × 10^5^) were infected with *H. pylori* (multiplicity of infection [MOI] = 200:1) for 2 h. The drug group was then pre-treated for 2 h. After 2 h, the culture was aspirated, 4% paraformaldehyde (Regen Biotechnology, Beijing, China) was added, incubated at room temperature for 20 min, paraformaldehyde was discarded, and the cells were washed thrice with PBST [PBS + 0.05% Tween 20 (Solarbio, Beijing, China)] and maintained for 5 min each time. After washing, PBST containing 0.1% Triton (Solarbio, Beijing, China) was added for 20 min to permeabilize the cells. Next, blocking solution [TBST containing 5% BSA (Lablead, Beijing, China)] was added for 30 min at room temperature, and the blocking solution was removed and cleaned twice with PBST. Then, the primary anti-Helicobacter pylori antibody (ab20459, Abcam, Cambridge, United Kingdom) was added and incubated in a wet box overnight at 4°C. The cells were washed three times with PBST and Alexa Fluor^®^ 488-labeled goat anti-rabbit IgG secondary antibody (Zhongshan Jinqiao Biotechnology, Beijing, China), incubated at room temperature, and protected from light for 1 h. After 1 h, the secondary antibody was aspirated, washed three times with PBST, and stained with mounting medium containing DAPI (Zhongshan Jinqiao Biotechnology, Beijing, China) for 5 min. The DAPI and fluorescein isothiocyanate (FITC) channels were selected under a fluorescence microscope (Nikon, Tokyo, Japan) and photographed for analysis. The results were expressed as *H. pylori* fluorescence area/DAPI fluorescence area calculated using ImageJ software.

### Statistical analysis

Data are presented as mean ± standard deviation. Differences between groups were assessed using one-way ANOVA. Pairwise comparisons were performed using Dunnett’s or Tukey’s *post hoc* test. *p* was set at *p* < 0.05. Statistical analysis was performed using the GraphPad Prism 8.1 software.

## Results

### Screening of drug-resistant *Helicobacter pylori* strains by E-test

According to EUCAST Clinical Breakpoint standard 2022, *H. pylori* strains with AML MIC > 0.125 μg/ml, CLR MIC > 0.5 μg/ml, LEV MIC > 1 μg/ml, and MTZ MIC > 8 μg/ml were antibiotic resistant. Seven drug-resistant *H. pylori* strains were screened, including six multidrug-resistant strains (Nos. 1–6) and one single drug-resistant strain (26695–16R). There were five CLR-resistant strains (Nos. 1, 2, 4, 5, and 6), five LEV-resistant strains (Nos. 1, 2, 3, 4, 6, and 26,695–16R), six MTZ-resistant strains (Nos. 1, 2, 3, 4, 6, and 26,695–16R), and one AML-resistant strain (No. 1). The MICs of the antibiotics against *H. pylori* are listed in Table 3.

**Table 3:** Minimum inhibitory concentration (MIC) of antibiotics against *H. pylori.*

*H. pylori* strains	MIC of antibiotics against *H. pylori* (μg/ml)			
	AML	CLR	LEV	MTZ
1	0.19^*^	32^*^	32^*^	256^*^
2	0.023	24^*^	32^*^	192^*^
3	0.016	0.016	32^*^	256^*^
4	0.016	32^*^	32^*^	256^*^
5	0.016	4^*^	32^*^	1.5
6	0.016	12^*^	0.25	256^*^
26,695–16R	0.016	0.016	0.25	64^*^

* Antibiotic resistance.

MIC, minimum inhibitory concentration; AML, amoxicillin; CLR, clarithromycin; LEV, levofloxacin; MTZ, metronidazole.

### *In vitro* antibacterial activities of JWC on *Helicobacter pylori*

The MICs of JWC against the drug-resistant strains were determined using the agar dilution method. The results showed that JWC had considerable antibacterial activity against drug-resistant *H. pylori* strains, with MIC values ranging from 64 to 1,024 μg/ml (Table 4), suggesting that there were differences in the antibacterial activity against different strains.

**Table 4:** MIC of JWC against *H. pylori.*

*H. pylori* strains	Concentration of JWC (μg/ml)											
	0	DMSO	4	8	16	32	64	128	256	512	1,024	2,048
1	+	+	+	+	+	+	+	−	−	−	−	−
2	+	+	+	+	+	+	+	−	−	−	−	−
3	+	+	+	+	+	+	+	−	−	−	−	−
4	+	+	+	+	+	+	−	−	−	−	−	−
5	+	+	+	+	+	+	+	+	+	+	−	−
6	+	+	+	+	+	+	−	−	−	−	−	−
26,695–16R	+	+	+	+	+	+	−	−	−	−	−	−

MIC, minimum inhibitory concentration; JWC, Jinghua Weikang capsule; +, existing colonies; −, no colony growth.

### The MICs of antibiotics after JWC intervention

JWC at ½ MIC was used to inhibit drug-resistant *H. pylori* strains. Based on previous studies (Hirata et al., 2010; Tsugawa et al., 2011), the concentration of PaβN used in this study was 20 μg/ml, which had no inhibitory effect on *H. pylori* 26,695–16R growth (Supplementary Table S1). Owing to the toxicity of CCCP, which had an obvious inhibitory effect on *H. pylori* 26,695–16R growth, its MIC against *H. pylori* 26,695–16R was determined to be 5 μg/ml using the agar dilution method (Supplementary Table S2); to exclude the bactericidal effect of CCCP itself, we used a concentration of 1 μg/ml for the test (Supplementary Figure S1). The results showed that JWC and efflux pump inhibitors PaβN (20 μg/ml) and CCCP (1 μg/ml) had no effect on the MICs of LEV-resistant strains but had a slight effect on the MICs of CLA-resistant strains. However, the MIC of MTZ against *H. pylori* 26,695–16R decreased from 64 μg/ml to 6 μg/ml after treatment with ½ MIC of JWC. The drug resistance of *H. pylori* 26,695–16R to MTZ was reversed, and its effect was better than that of efflux pump inhibitors PaβN (20 μg/ml) and CCCP (1 μg/ml; Tables 5–7).

**Table 5:** Effects of JWC on MIC (μg/ml) of LEV resistant strains.

*H. pylori* strains	Con	PaβN	CCCP	JWC
1	32	32	32	32
2	32	32	32	32
3	32	32	32	32
4	32	32	32	32
5	32	32	32	32

**Table 6:** Effects of JWC on MIC (μg/ml) of CLA resistant strains.

*H. pylori* strains	Con	PaβN	CCCP	JWC
1	32	32	32	32
2	24	24	24	24
4	32	32	32	32
5	4	2	4	4
6	12	4	8	8

**Table 7:** Effects of JWC on MIC (μg/ml) of MTZ resistant strains.

*H. pylori* strains	Con	PaβN	CCCP	JWC
1	256	256	256	256
2	192	192	192	192
3	256	256	256	256
4	256	256	256	256
6	256	256	256	256
**26,695–16R**	**64**	**48**	**24**	**6**

MIC, minimum inhibitory concentration; JWC, Jinghua Weikang Capsule; CLR, clarithromycin; LEV, levofloxacin; MTZ, metronidazole.

In this study, JWC had different MICs for different drug-resistant *H. pylori* strains and showed a unique effect on reducing MTZ resistance in *H. pylori* 26,695–16R, suggesting that its effect on reducing drug resistance may also be affected by bacterial characteristics. To further explore the possible mechanism based on the confirmed effect, *H. pylori* 26,695–16R was selected as the research object in subsequent experiments.

### Inhibiting kinetics and killing kinetics assay

**Figure 1:**
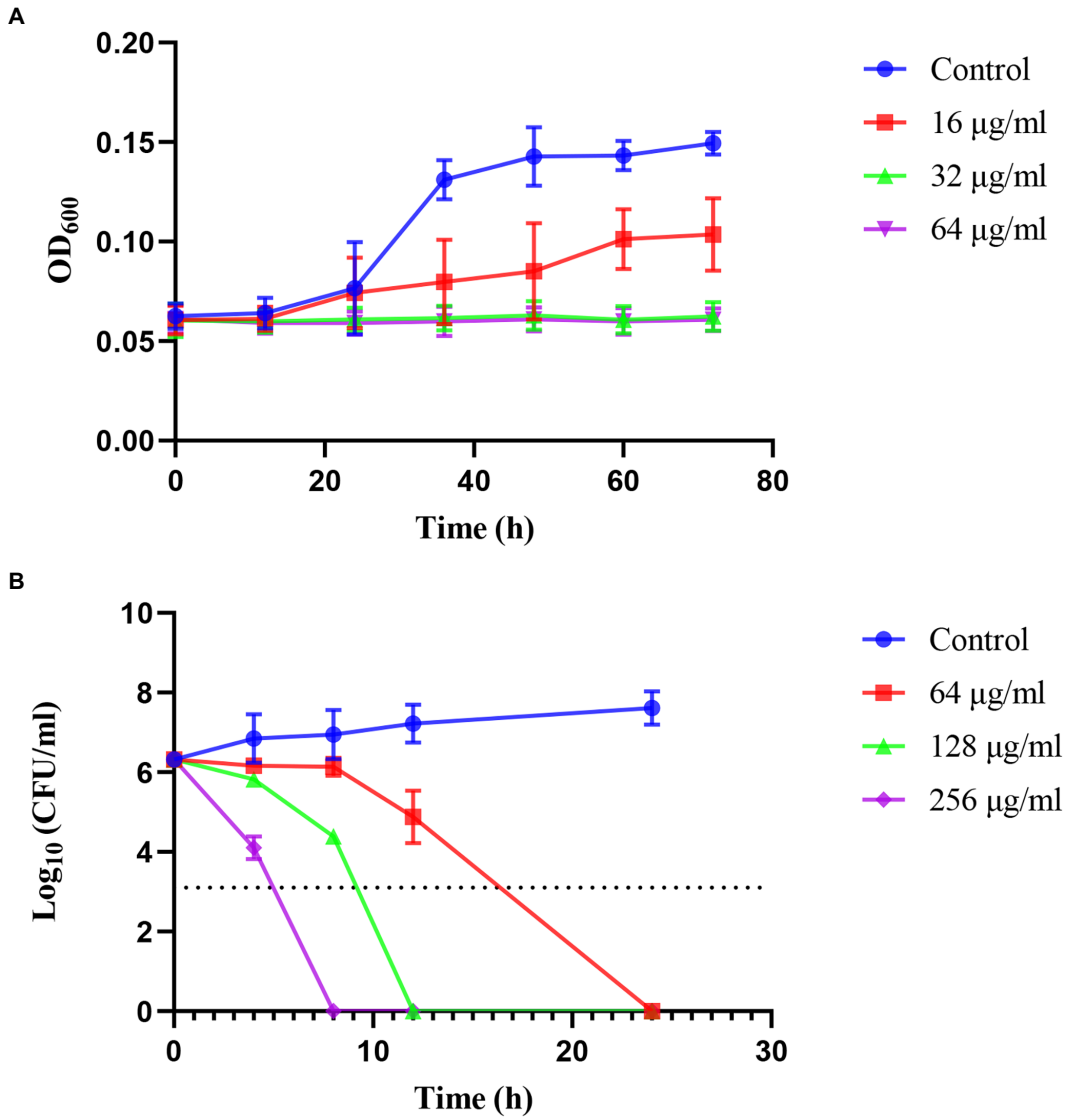
Inhibiting kinetic curves and killing kinetic curves. **(A)** Inhibiting kinetics curves of JWC on *Helicobacter pylori* 26,695–16R. **(B)** Killing kinetics curves of JWC on *H. pylori* 26,695–16R. The dotted line represents a 1,000-fold reduction in the number of bacteria compared to the initial inoculation.

The kinetics of the inhibition and killing of *H. pylori* 26,695–16R by JWC were time-and dose-dependent (Figure 1). JWC inhibited the growth of *H. pylori* 26,695–16R at concentrations as low as 16 μg/ml (1/4 MIC). The OD_600_ of the bacterial solution did not increase significantly after treatment with 32 μg/ml (1/2 MIC) and 64 μg/ml (MIC) concentrations of JWC (Figure 1A). JWC killed *H. pylori* 26,695–16R at 64–256 μg/ml (MIC to 4 MIC), which indicated a 1,000-fold reduction in the number of bacteria compared with the initial inoculation. JWC at 64–256 μg/ml (MIC to 4 MIC) completely killed *H. pylori* 26,695–16R after 8–24 h of intervention (Figure 1B).

### Detection of drug resistance-related genes in *Helicobacter pylori* 26,695–16R

**Figure 2:**
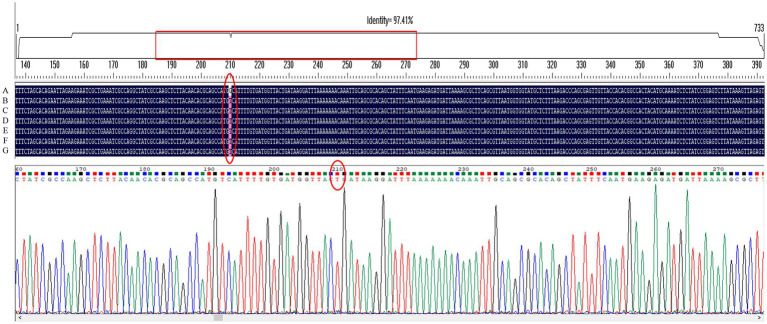
G210T point mutation in the MTZ resistance-related gene *rdxA* of *H. pylori* 26,695–16R under different drug interventions. A, *H. pylori* 26,695; B, *H. pylori* 26,695–16R; C, Treated with PaβN (20 μg/ml); D, Treated with CCCP (1 μg/ml); E, Treated with JWC 16 μg/ml; F, Treated with JWC 32 μg/ml; G, Treated with JWC 64 μg/ml.

Gene sequencing results showed that only the G210T point mutation occurred in the MTZ resistance-related gene *rdxA* among several genes detected in *H. pylori* 26,695–16R, and intervention with the efflux pump inhibitors PaβN (20 μg/ml), CCCP (1 μg/ml), and JWC did not affect the mutation, as shown by the red ellipse in Figure 2. Primers used in these experiments are listed in Table 1.

### Effect of JWC on the biofilm of *Helicobacter pylori* 26,695–16R by crystal violet method

**Figure 3:**
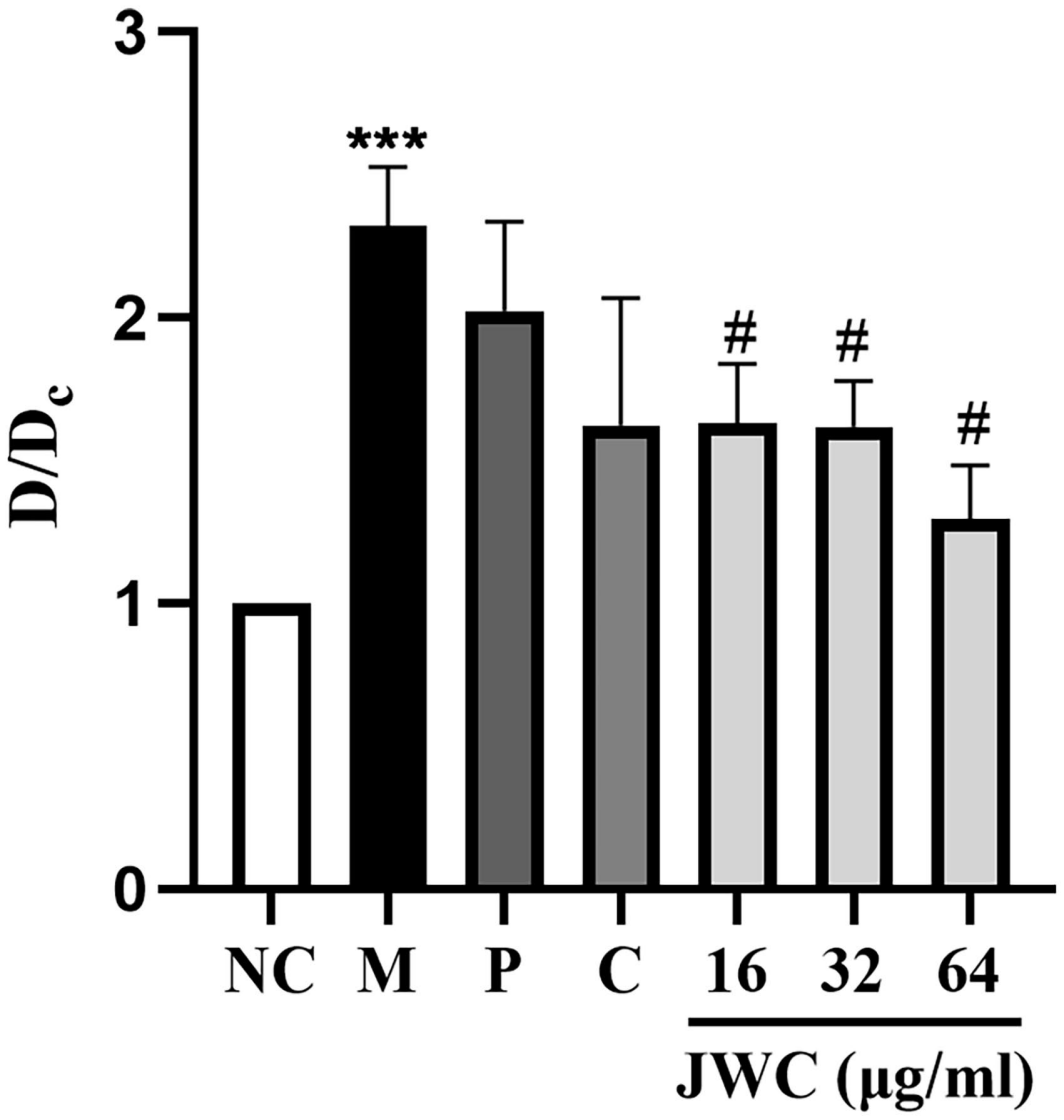
Effect of JWC, efflux pump inhibitors and MTZ on the biofilm of *H. pylori* 26,695–16R. Dc is the OD value of the blank control group, and D is the OD value of the other groups. NC: the blank control group, culture medium without *H. pylori* 26,695–16R; M, Model group, culture medium with *H. pylori* 26,695–16R; P, PaβN (20 μg/ml); C, CCCP (1 μg/ml). ^***^p < 0.001, compared with the results of the control group. ^#^*p* < 0.05, compared with the results of the model group.

When D/Dc > 4, it was determined to be a strong biofilm-forming strain; 2 < D/Dc ≤ 4, a moderate biofilm-forming strain; and 1 < D/Dc ≤ 2, a weak biofilm-forming strain. D/Dc ≤ 1 was defined as a strain without biofilm formation. The results showed that *H. pylori* 26,695–16R is a moderate biofilm-forming strain, and CCCP (1 μg/ml) and JWC (16–64 μg/ml) inhibited biofilm formation in *H. pylori* 26,695–16R, and the difference was statistically significant (Figure 3).

### Effect of JWC on the biofilm of *Helicobacter pylori* 26,695–16R by SEM

**Figure 4:**
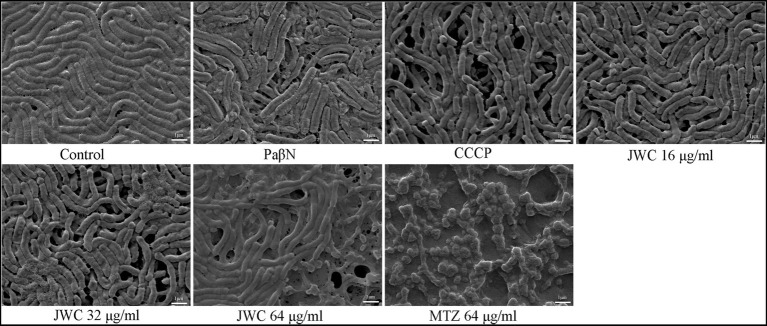
Effect of JWC and efflux pump inhibitors on the biofilm of *H. pylori* 26,695–16R.

The normal group showed a biofilm structure formed by *H. pylori* 26,695–16R. Bacteria and extracellular matrix that are closely linked to bacteria can be found in biofilms. The bacteria were mostly rod-shaped with tight connections and fewer voids. After treatment with CCCP (1 μg/ml) and JWC, the biofilm structure of bacteria was disrupted, connections between bacteria became sparse, and the number of voids increased. The degree of destruction of the bacterial biofilm structure increased with increasing JWC concentrations. After treatment with MTZ at a concentration of 64 μg/ml, *H. pylori* 26,695–16R cells converted into a coccoid shape, which is a stress response to MTZ and one of the survival mechanisms of *H. pylori* (Figure 4).

### Effect of JWC on the biofilm of *Helicobacter pylori* 26,695–16R by CLSM

**Figure 5:**
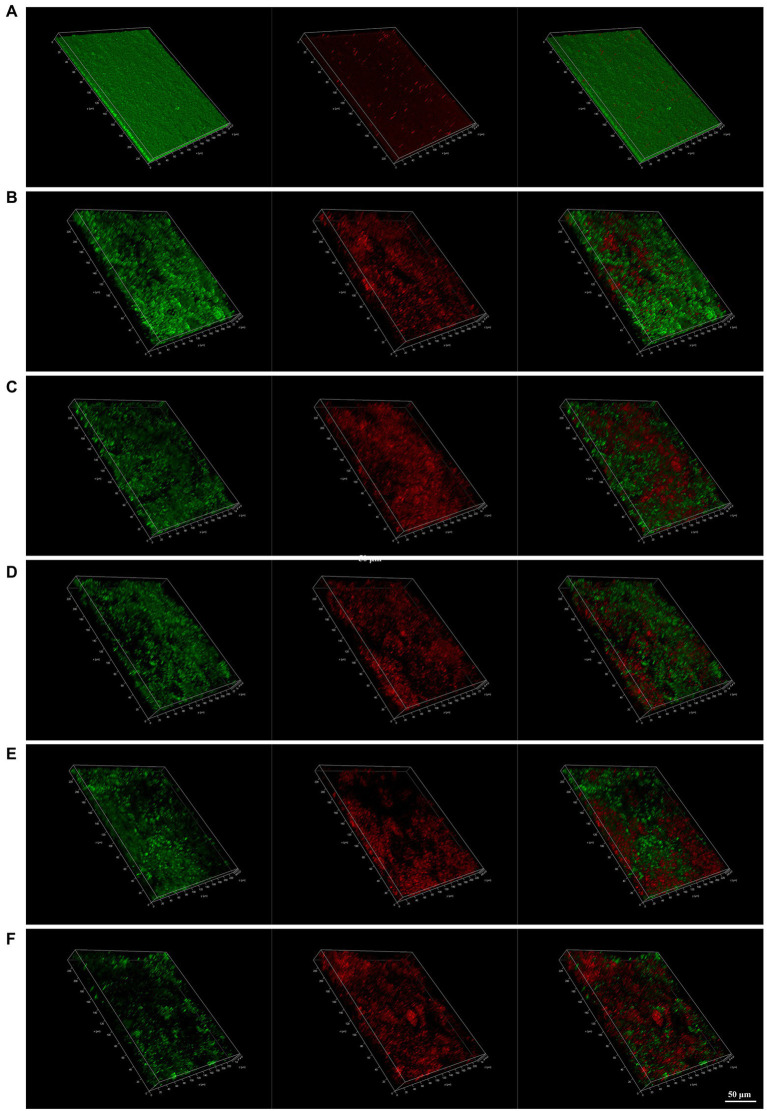
Confocal laser scanning microscope (CLSM) images of *H. pylori* 26,695–16R strain biofilms. Cells stained with membrane-permeant SYTO 9 (green) and membrane-impermeant propidium iodide (red) were visualized by CLSM. **(A)** Control group; **(B)** treated with PaβN (20 μg/ml); **(C)** treated with CCCP (1 μg/ml); **(D)** treated with JWC 16 μg/ml; **(E)** treated with JWC 32 μg/ml; **(F)** treated with JWC 64 μg/ml.

CLSM images provide a rough outline of *H. pylori* biofilms and the viability of the bacteria. Green represents live bacteria and red represents dead bacteria. In the control group, we observed that *H. pylori* 26,695–16R formed a dense biofilm of a certain thickness and good bacterial activity. After treatment with PaβN (20 μg/ml), CCCP (1 μg/ml), and JWC (16–64 μg/ml), the biofilm structure of *H. pylori* 26,695–16R loosened, with a reduced proportion of live bacteria and an increased proportion of dead bacteria, indicating that biofilm formation was inhibited. When the concentration of JWC increased, the ratio of live/dead bacteria decreased and the amount of biofilm formed by bacterial aggregation decreased (Figure 5).

### Influence of JWC on efflux pump gene expression in *Helicobacter pylori* 26,695–16R

**Figure 6:**
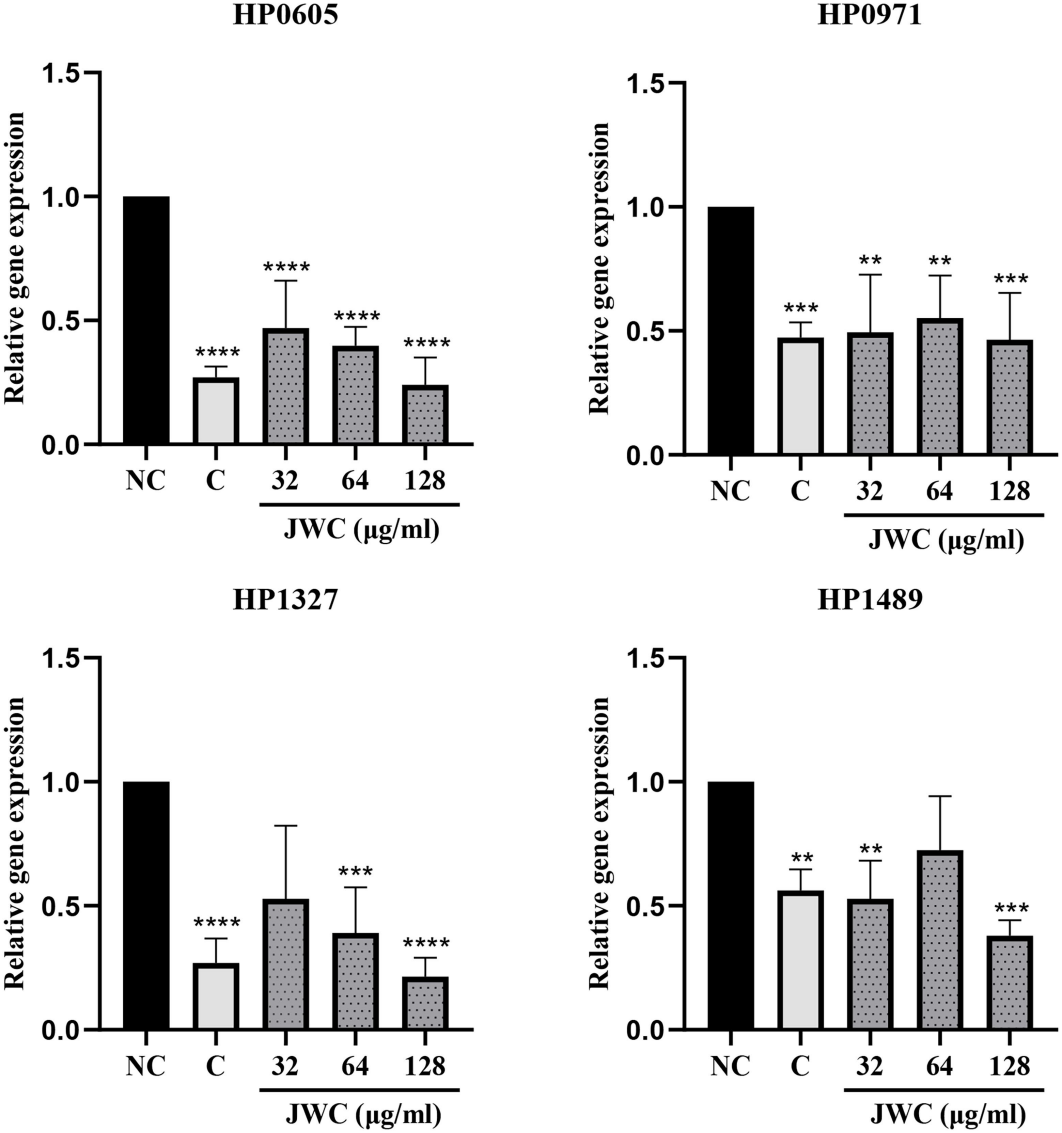
Expression of different efflux effect genes of *H. pylori* 26,695–16R after incubation with PaβN, CCCP and different concentrations of JWC. NC, Normal control group; C, CCCP (1 μg/ml); ^**^p < 0.01, ^***^p < 0.001, ^****^p < 0.0001, compared with the results of the control group.

qPCR results showed that both CCCP and JWC inhibited the expression of HP0605 and HP0971. CCCP and JWC at 64 μg/ml (MIC) and 128 μg/ml (2 MIC) inhibited the expression of HP1327. CCCP and JWC at 32 μg/ml (1/2 MIC) and 128 μg/ml (2 MIC) inhibited the expression of HP1489, and the difference was statistically significant, as shown in Figure 6. The primers used are listed in Table 2.

### Jinghua Weikang capsule inhibited the adhesion of *Helicobacter pylori* 26,695–16R to GES-1 cells

**Figure 7:**
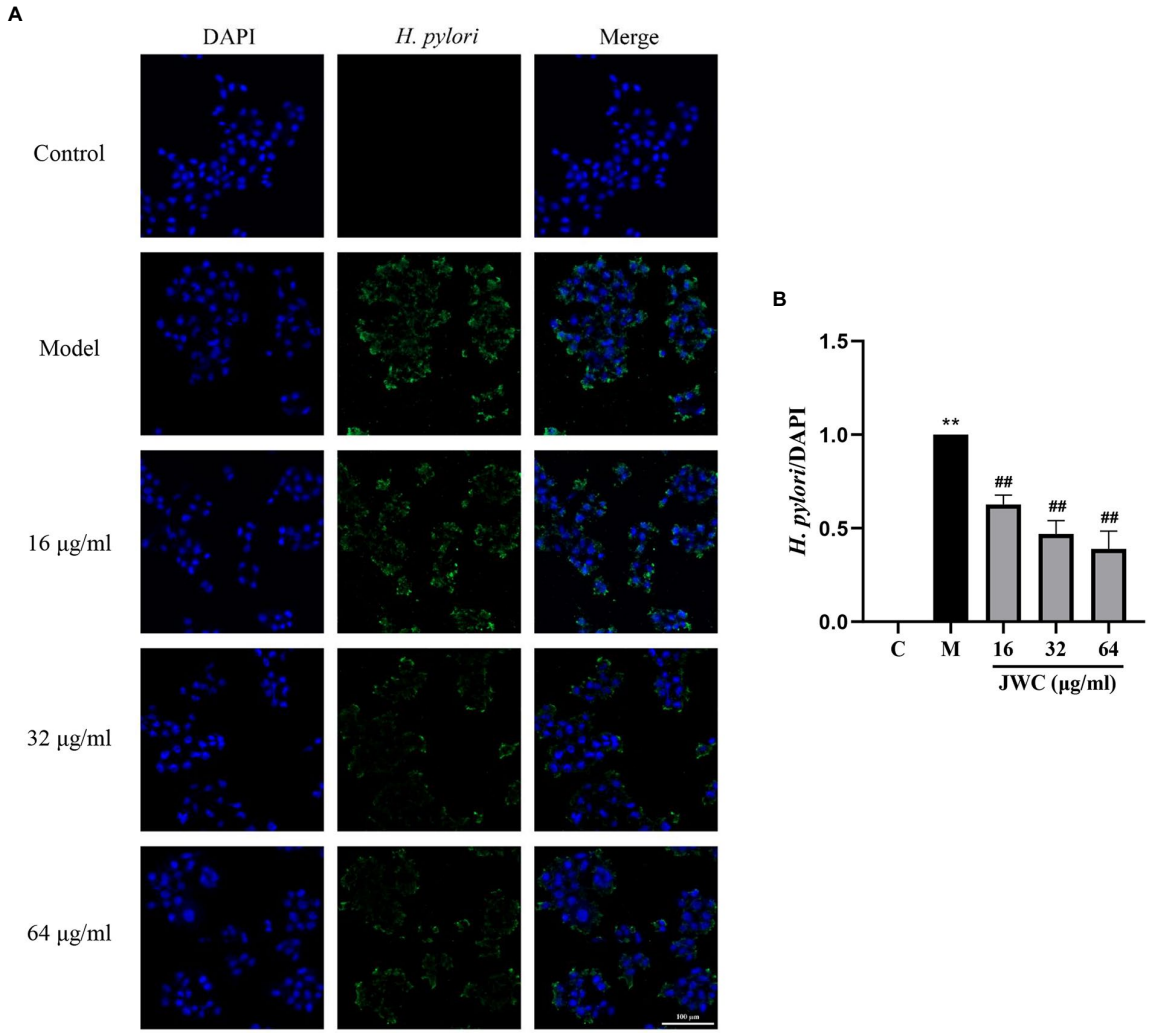
Effect of JWC on the adhesion of *H. pylori* 26,695–16R to GES-1 cells. **(A)** Immunofluorescence images of *H. pylori* and GES-1 adhesion after JWC intervention. Control group, only GES-1 cells; Model group, GES-1 and *H. pylori*. **(B)** The *H. pylori* fluorescence area/DAPI fluorescence area calculated by ImageJ software. ^**^p < 0.01, compared with the results of the control group. ^##^*p* < 0.01, compared with the results of the model group. Bold values represent a meaningful MIC change of antibiotic and the *H. pylori* strain used in subsequent experiments.

As shown in Figure 7, blue fluorescence indicates the nucleus of GES-1 cells and green fluorescence indicates *H. pylori*. After observing the pictures in different channels, they were merged using a filter function. The merged results showed that *H. pylori* can adhere to GES-1 cells and that 16, 32, and 64 μg/ml JWC dose-dependently reduced the amount of *H. pylori* adherence (Figure 7A). According to the results of fluorescence intensity analysis using Image J software, the adhesion of *H. pylori* was inhibited by 40–60% when 16–64 μg/ml JWC was used (Figure 7B).

### Effect of JWC on adhesins of *Helicobacter pylori* 26,695–16R

**Figure 8:**
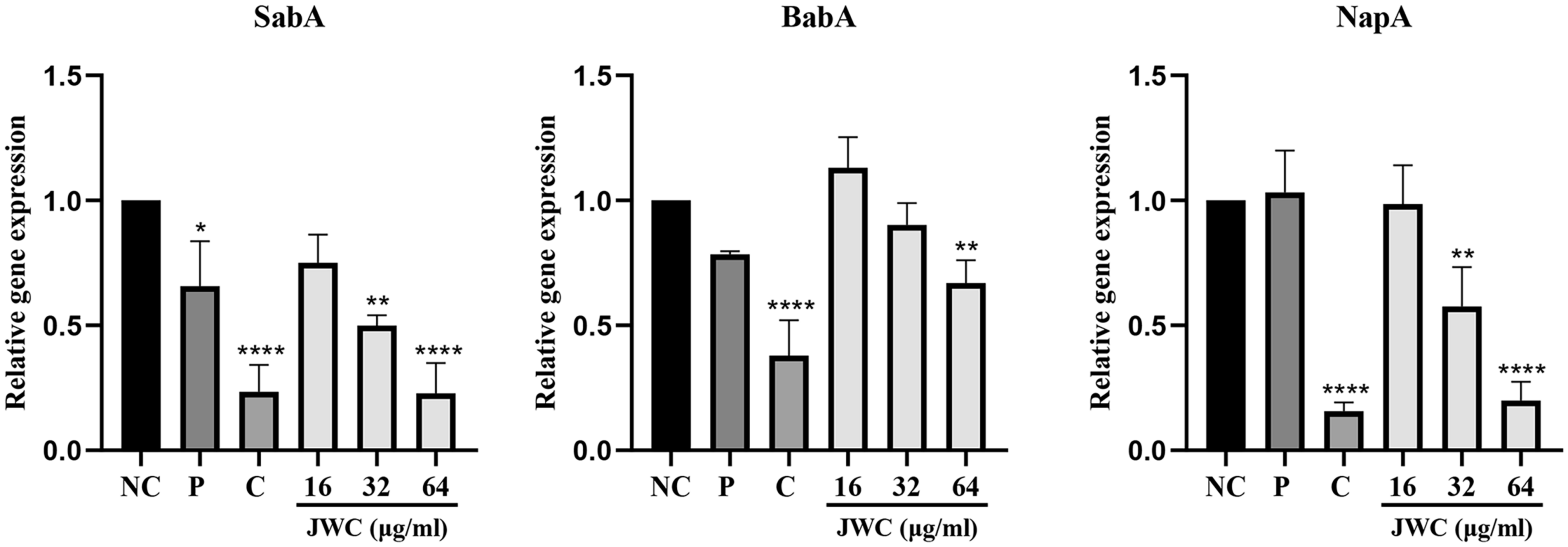
Effect of JWC on adhesions of *H. pylori* 26,695–16R. NC, Normal control group, *H. pylori* 26,695–16R; P, PaβN (20 μg/ml); C, CCCP (1 μg/ml). ^*^p < 0.05, ^**^p < 0.01, ^****^p < 0.0001, compared with the results of the control group.

The results showed that 32 μg/ml and 64 μg/ml JWC and CCCP decreased the expression of *SabA*, *BabA* and *NapA*. PaβN only decreased *SabA*. On the whole. JWC decreased the expression of adhesins in a concentration-dependent manner (Figure 8).

## Discussion

Antibiotic resistance of *H. pylori* has been increasing worldwide (Tshibangu-Kabamba and Yamaoka, 2021; Ho et al., 2022; Veenendaal et al., 2022; Zhong et al., 2022). Resistance of *H. pylori* to MTZ is more common than that of other antibiotics (Gerrits et al., 2006; Ho et al., 2022). MTZ resistance rates range from 42 to 96% worldwide, with higher rates in developing countries (Geng et al., 2022; Ho et al., 2022; Liu et al., 2022; Tian et al., 2022; Veenendaal et al., 2022). In triple, quadruple, and sequential therapies, including MTZ, eradication failure is often associated with MTZ resistance (Fischbach and Evans, 2007; Gatta et al., 2009). The nitro moiety of MTZ is reduced to a highly active compound that exerts antibacterial activity against *H. pylori* (Jenks and Edwards, 2002). The inactivation of *rdxA* (which encodes an oxygen-insensitive NADPH nitroreductase), *frxA* (which encodes NADPH flavin oxidoreductase), and *fdxB* (which encodes a ferredoxin-like protein) was closely associated with the failure of enzymatic reduction and MTZ resistance (Jenks and Edwards, 2002). In this study, seven resistant strains were screened, and JWC was found to have a significant anti-drug resistance effect on the MTZ-resistant strain *H. pylori* 26,695–16R, reducing its MIC value against MTZ from 64 μg/ml to 6 μg/ml and reversing resistance. For *H. pylori* 26,695–16R, the effect of JWC on reducing MTZ resistance was better than that of the efflux pump inhibitors PaβN and CCCP. The G210T point mutation in the *rdxA* gene of *H. pylori* 26,695–16R was found by sequencing, but the point mutation did not change after drug treatment, suggesting that the mechanism by which JWC reverses MTZ resistance is not related to the *rdxA* mutation, and that JWC may influence MTZ resistance through other mechanisms.

In addition to gene mutations, biofilm formation *in vivo* is an important mechanism leading to drug resistance. Biofilms are communities of microorganisms attached to a surface, and the surrounding EPS matrix is composed of extracellular polysaccharides, DNA, and proteins (Rabin et al., 2015). Biofilms play an important role in the persistence of bacterial infections, reducing bacterial susceptibility to antibiotics, and counteracting host immune mechanisms, allowing bacteria to survive in hostile environments (Rabin et al., 2015; Del Pozo, 2018). In the initial stage of biofilm formation, *H. pylori* is helical, and after effective adhesion and proliferation on the surface, the morphology changes to helical, rod-shaped, curved, spherical, and filamentous. However, in prolonged culture, all cells in the biofilm eventually transformed into globular cells, indicating that they were involved in survival and had a higher tolerance to adverse environmental factors (Krzyżek et al., 2020). The crystal violet method showed that *H. pylori* 26,695–16R is a moderate biofilm-forming strain, and CCCP and JWC (16–64 μg/ml) could inhibit biofilm formation in *H. pylori* 26,695–16R. After treatment with CCCP and JWC, the biofilm structure of bacteria was disrupted, connections between bacteria became sparse, and the number of voids increased. The degree of destruction of the bacterial biofilm structure increased with increasing JWC concentrations. However, after treatment with MTZ at a concentration of 64 μg/ml, the morphology of *H. pylori* 26,695–16R became spherical, indicating that MTZ is a powerful toxic substance to bacteria, and that bacteria obtain stronger self-protection ability through sphericity, which is also the reason for their resistance to MTZ.

The efflux pump system is one of the mechanisms of biofilm formation and is a key nonspecific mechanism of drug resistance in gram-negative bacteria (Hall and Mah, 2017). The efflux pump expels toxic substances such as antibiotics from the bacterial cytosol, thereby reducing the intracellular concentration of antibiotics and conferring antibiotic resistance to the bacteria (Poole, 2007). The RND family, the most studies in regards to their involvement in bacterial biofilm formation, is composed of inner membrane, periplasmic membrane fusion, and outer membrane proteins (Nikaido, 2011). At least one efflux pump, AcrAB-TolC, belongs to the RND family, and four gene clusters encoding RND, namely, HP0605-HP0607 (*hefABC*), HP0971-HP0969 (*hefDEF*), HP1327-HP1329 (*hefGHI*), and HP1489-HP1487, are currently detected in *H. pylori* (Ye et al., 2020). An enhanced efflux system is the first step in the development of MTZ resistance in *H. pylori* (Tsugawa et al., 2011). Several studies have shown that the expression of the efflux pump gene in biofilm-forming cells was significantly higher than that in planktonic cells (Soto, 2013; Attaran et al., 2017). For example, the expression levels of HP0605-HP0607 (*hefABC*), HP0971-HP0969 (*hefDEF*), HP1327-HP1329 (*hefGHI*), and HP1489-HP1487 in biofilm-forming strains are higher than those in planktonic bacteria (Yonezawa et al., 2019). This study found that JWC reduced the expression of HP0605-HP0607 (*hefABC*), HP0971-HP0969 (*hefDEF*), HP1327-HP1329 (*hefGHI*), and HP1489-HP1487, suggesting that JWC may reduce *H. pylori* resistance to MTZ by reducing the expression of efflux pump genes, and may also indirectly affect biofilm formation by reducing the expression of efflux pump genes to reduce *H. pylori* resistance to MTZ. However, the specific mechanism by which efflux pumps affect biofilm formation is not clear at present, which may be related to the pumping of substances related to biofilm formation. A previous study found that emodin, baicalin, schizandrin, and berberine significantly decreased the MIC of amoxicillin and tetracycline against some *H. pylori* strains, and the mechanism may be related to the reduction in *hefA* mRNA expression (Huang et al., 2015). Our study is consistent with this view and complements the study of efflux pump genes and related biofilms.

Adhesion is the first step and prerequisite for biofilm formation. The expression of adhesins increases during the transition from the planktonic to the biofilm phase (Krzyżek et al., 2020). Several studies have shown that adhesive proteins, such as *NapA*, *AlpB*, *SabA*, *BabA*, *Homb*, *LabA* and *HopZ* are involved in biofilm formation (Cooksley et al., 2003; Yang et al., 2011; Acio-Pizzarello et al., 2017; Servetas et al., 2018; Zhao et al., 2021). Compared to wild-type strains, ArsRS mutants had high surface attachment and biofilm production, and the expression of genes encoding outer membrane proteins was increased in these mutants, including *AlpB*, *SabA*, *BabA*, *Homb*, *LabA* and *HopZ* (Acio-Pizzarello et al., 2017; Servetas et al., 2018). *NapA* is a surface protein that attracts and activates neutrophils and promotes endothelial adhesion and production of oxygen radicals and chemokines (D’Elios et al., 2007). When Helicobacter pylori is exposed to oxidative stress, *NapA* is highly expressed to resist oxygen stress injury, thus relieving bacterial survival pressure and promoting the formation and aggregation of EPS to promote biofilm formation (Cooksley et al., 2003; Zhao et al., 2021). Another study also showed that *NapA* plays a role in adhesion to a substratum and *H. pylori* and hence influences biofilm formation (Yang et al., 2011). In this study, we found that JWC dose-dependently reduced the adhesion of *H. pylori* to GES-1 cells and the expression of adhesives *NapA*, *sabA* and *babA*, suggesting that JWC may reduce the adhesion between *H. pylori* and *H. pylori* or GES-1 by decreasing the expression of adhesins, thus affecting the formation of biofilms and inhibiting drug resistance.

Although it was found in this study that JWC considerably reduced the drug resistance of MTZ-resistant strain 26,695–16R, it did not reduce the drug resistance of other strong MTZ-resistant strains, indicating that the ability of JWC to reduce MTZ resistance is not universal and may be related to the strength of MTZ resistance of the strain itself. The reversal of metronidazole resistance by JWC may be achieved through the adhesin/RND efflux pump-biofilm pathway.

## Conclusion and outlook

JWC had good antibacterial effects against drug-resistant *H. pylori* strains and reversed the drug resistance of the MTZ-resistant strain 26,695–16R *in vitro*, the mechanism of which was related to the adhesin/RND efflux pump-biofilm pathway. However, biofilms are difficult to construct in animals because of the low ratophilic nature of *H. pylori*, which requires further experimental exploration *in vivo*. The mechanisms by which efflux pumps and adhesins inhibit biofilm formation require further investigation. In addition to its bactericidal effect, JWC has the advantages of reducing drug resistance and multi-targeting, reflecting the concept of potentiation rather than pure antagonism against drug-resistant *H. pylori*. This study provides an explanation for the mechanism by which JWC inhibits drug-resistant *H. pylori*, experimental support for the clinical application of JWC in combination with triple or quadruple therapy, and ideas for the clinical treatment of *H. pylori* and the development of new drugs.

## Data availability statement

The raw data supporting the conclusions of this article will be made available by the authors, without undue reservation.

## Author contributions

XJ performed the experiments, wrote the manuscript, and summarized and analyzed the data. QH and ML participated in performing the experiments. HY and ZS revised the manuscript. YC participated in the data analysis. HY designed the study. XZ and HY finally reviewed and approved the article for publication. All authors contributed to the article and approved the submitted version.

## Funding

The study is supported by the National Science Foundation of China (nos. 81803910 and 81973615) and Qi-Huang Scholar Chief Scientist Program of National Administration of Traditional Chinese Medicine Leading Talents Support Program (2021).

## Conflict of interest

The authors declare that the research was conducted in the absence of any commercial or financial relationships that could be construed as a potential conflict of interest.

## Publisher’s note

All claims expressed in this article are solely those of the authors and do not necessarily represent those of their affiliated organizations, or those of the publisher, the editors and the reviewers. Any product that may be evaluated in this article, or claim that may be made by its manufacturer, is not guaranteed or endorsed by the publisher.
