# Supplementary material for: Revealing the novel effect of Jinghua Weikang capsule against the antibiotic resistance of Helicobacter pylori
Source: Front Microbiol. 2022 Sep 6;13:962354. doi: 10.3389/fmicb.2022.962354 (PMC9485998; doi:10.3389/fmicb.2022.962354)
Supplement: Supplementary file 1 [file Data_Sheet_1.DOCX]

Supplementary Material

PaβN (10–40 μg/ml) did not inhibit *H. pylori* 26695-16R growth (Table S1). Owing to CCCP’s toxicity, which has a inhibitory effect on *H. pylori* 26695-16R growth, its MIC against *H. pylori* 26695-16R was determined to be 5 μg/ml using the agar dilution method (Table S2). CCCP partially inhibited the growth of *H. pylori* 26695-16R at 2.5 μg/ml and had no effect on the growth of *H. pylori* 26695-16R at 1 μg/ml (Fig S1).

**Table S1** MIC of PaβN against *H. pylori*

| *H. pylori* Strains | [Concentration](D:/%E5%BA%94%E7%94%A8/Dict/8.9.9.0/resultui/html/index.html#/javascript:;) of PaβN (μg/ml) | | | | | |
| --- | --- | --- | --- | --- | --- | --- |
|  | 0 | 2.5 | 5 | 10 | 20 | 40 |
| 26695-16R | 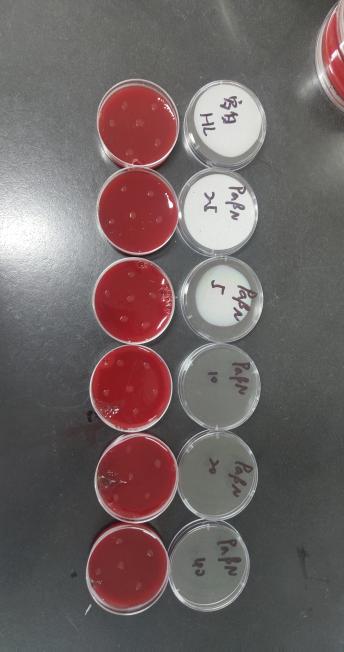+ | + | + | + | + | + |
|  |  |  |  |  |  |  |

MIC: minimum inhibitory concentration; PaβN: phenylalanine-arginine β-naphthylamide.

**Table S2** MIC of CCCP against H. pylori

| *H. pylori* Strains | [Concentration](D:/%E5%BA%94%E7%94%A8/Dict/8.9.9.0/resultui/html/index.html#/javascript:;) of CCCP (μg/ml) | | | | | | | |
| --- | --- | --- | --- | --- | --- | --- | --- | --- |
|  | 0 | DMSO | 1 | 2.5 | 5 | 10 | 20 | 40 |
| 26695-16R | 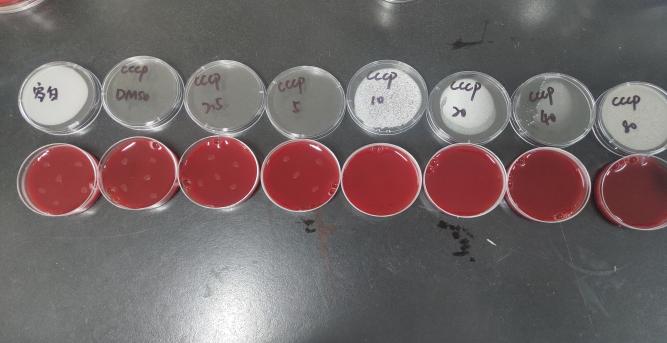+ | + | + | + | + | − | − | − |
|  |  |  |  |  |  |  |  |  |

MIC: minimum inhibitory concentration; CCCP: carbonyl cyanide m-chlorophenylhydrazonequinoline


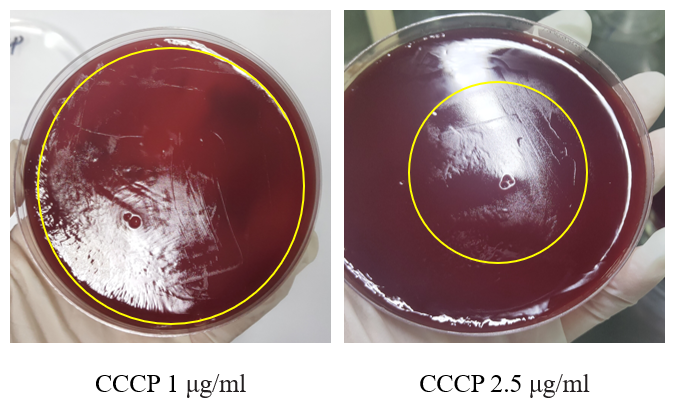


**Fig S1** Effects of different concentrations of CCCP on *H. pylori* 26695-16R growth. The yellow circles represent the growth regions of *H. pylori* in response to different concentrations of CCCP.
